# The Landscape of the Genomic Distribution and the Expression of the F-Box Genes Unveil Genome Plasticity in Hexaploid Wheat during Grain Development and in Response to Heat and Drought Stress

**DOI:** 10.3390/ijms22063111

**Published:** 2021-03-18

**Authors:** Claire Guérin, Saïd Mouzeyar, Jane Roche

**Affiliations:** UMR 1095 Génétique, Diversité et Ecophysiologie des Céréales, Université Clermont Auvergne, INRAE, 63000 Clermont-Ferrand, France; claire.guerin@uca.fr (C.G.); said.mouzeyar@uca.fr (S.M.)

**Keywords:** FBX, wheat, family expansion, small-scale duplication, heat stress, drought

## Abstract

FBX proteins are subunits of the SCF complex (Skp1–cullin–FBX) belonging to the E3 ligase family, which is involved in the ubiquitin–proteasome 26S (UPS) pathway responsible for the post-translational protein turnover. By targeting, in a selective manner, key regulatory proteins for ubiquitination and 26S proteasome degradation, FBX proteins play a major role in plant responses to diverse developmental and stress conditions. Although studies on the genomic organization of the *FBX* gene family in various species have been reported, knowledge related to bread wheat (*Triticum aestivum*) is scarce and needs to be broadened. Using the latest assembly of the wheat genome, we identified 3670 *TaFBX* genes distributed non-homogeneously within the three subgenomes (A, B and D) and between the 21 chromosomes, establishing it as one of the richest gene families among plant species. Based on the presence of the five different chromosomal regions previously identified, the present study focused on the genomic distribution of the *TaFBX* family and the identification of differentially expressed genes during the embryogenesis stages and in response to heat and drought stress. Most of the time, when comparing the expected number of genes (taking into account the formal gene distribution on the entire wheat genome), the *TaFBX* family harbors a different pattern at the various stratum of observation (subgenome, chromosome, chromosomal regions). We report here that the local gene expansion of the *TaFBX* family must be the consequence of multiple and complex events, including tandem and small-scale duplications. Regarding the differentially expressed *TaFBX* genes, while the majority of the genes are localized in the distal chromosomal regions (R1 and R3), differentially expressed genes are more present in the interstitial regions (R2a and R2b) than expected, which could be an indication of the preservation of major genes in those specific chromosomal regions.

## 1. Introduction

During their growth, and in response to external stimuli, plants continuously adapt through fundamental cellular activities. In these molecular mechanisms, cellular protein degradation and recycling processes often occur and are regulated by different conserved mechanisms in all eukaryotes [1]. One of these mechanisms is the Ubiquitin–proteasome system (UPS), an ATP-dependent highly regulated system that ensures the degradation of short-lived transcription factors, damaged or misfolded proteins and other regulatory proteins [2,3,4].

The UPS acts through a two-step-process involving the poly-ubiquitination of the targeted protein and the 26S proteasome-mediated degradation. Target protein ubiquitination is mediated by the sequential action of three enzymes, E1 (ubiquitin-activating enzyme), E2 (ubiquitin-conjugating enzyme), and E3 (ubiquitin ligases). Only a few E1 and E2 enzymes have been identified in plants (2 and 6 E1, and 47 and 49 E2, in Arabidopsis and rice, respectively), whereas 1305 and 1332 E3 ligases have been identified in these same species [5]. Among the E3 ligases, some are multimeric complexes, such as the SCF complex, which is composed of four major subunits: Skp1, Cul/Cdc53, Roc1/Rbx1/Hrt1 and an F-box protein (FBX). The FBX protein provides specificity through recognition of the substrate to be ubiquitinated [4]. The high number of FBX subunits in plants may reflect their involvement in numerous, diverse and core molecular processes related to cell cycle development, hormonal or environmental stress responses [6,7,8,9,10,11,12,13,14,15,16,17,18,19,20,21,22,23].

FBX proteins are defined by an F-box domain signature located in the N-terminal region and dedicated to the binding of the protein to an SKP protein [24,25,26]. The FBX also carries a more variable and multi-domain C-terminal region related to its role in recognition of different substrates [27]. The C-terminal motifs have been used to classify FBX proteins into different subfamilies. For example, Jin et al. (2004) identified 69 human FBX classified into three groups, according to their domains (LRRs, WD40 repeats and other domains) [28].

The FBX family has been recorded in a large number of animal and plant species [5]; for example, 9 in humans, 74 in mice, 20 in yeast and 27 FBX in *Drosophila* [28,29]. In plants, the FBX family is much larger, with 897 predicted genes in *Arabidopsis*, 971 in rice, 417 in corn, 228 in *Physcomitrella patens* moss, 285 in chickpea, 592 in cotton and 425 genes in poplar [25,30,31,32]. In contrast to other E3 ligases families, the FBX family has, therefore, undergone a significant expansion in the plant kingdom in comparison with other eukaryotes [33,34]. The emergence and expansion of gene families and subsequent functional divergence among family members have been hypothesized to be the principal driving forces in the adaptation of organisms to different environments [35,36]. In wheat, other gene families, such as transcription factors (for example, MAD-box containing 201 genes [37] or TaNAC family containing 488 genes [38]), have been submitted to gene expansion phenomena through tandem duplication retrotransposition or large-scale duplications of the inter and intra-chromosomal regions. Plants appear to have exploited multiple gene families to adapt to numerous developmental and physiological processes. These families arose, expanded and diversified through whole-genome duplications (polyploidy), which are common in many plant lineages, more restricted segmental duplications, highly specific tandem duplications and transposon-mediated events and even exon shuffling among individual genes [39,40,41,42]. Until very recently, a complete inventory of the FBX genes was missing in bread wheat, one of the most important cereal crop species. In 2020, 1796 *TaFBX* genes were identified and classified into subfamilies [43]. Meanwhile, using the latest version of the wheat genome, we investigated the same *TaFBX* gene inventory. Our results lead to a larger number of *TaFBX* genes (3670), suggesting a plethora of duplication events that could have occurred through the evolutionary history of wheat and that are discussed in this article. In addition, using publicly available RNAseq data [44], the expression profile of the 3670 genes was monitored in two different physiological situations (embryogenesis/grain development and in response to three abiotic stress (heat, drought and a combination of both)); and the differentially expressed genes (DEGs) were localized in five chromosomal regions of the 21 wheat chromosomes. In this paper, our results also show that both the chromosomal distribution of the F-box coding genes and their expression are not evenly distributed, suggesting that the interstitial regions of the chromosomes are more involved than the distal regions.

## 2. Results

### 2.1. Structure, Organization and Genomic Distribution of the TaFBX Family in Bread Wheat

#### 2.1.1. Identification of Wheat TaFBX Gene Family

The hidden Markov models (HMM) profile of the Pfam F-box domain and FBX-like domains (PF00646, PF12937 and PF13013, PF15966) were used as queries to identify *TaFBX* genes in the RefSeq v1.1 version of the annotated wheat genome released by the International Wheat Genome Sequencing Consortium (IWGSC) [45]. This first query led to the identification of 4303 putative *TaFBX* genes. InterProScan analysis and manual curation of domain organization consistency eliminated 633 sequences and left 3670 full-length, high-confidence (HC) sequences containing an F-box or an F-box-like domain (Table S1). Among them, 3536 (96.3%) *TaFBX* genes were mapped onto the 21 bread wheat chromosomes; the 134 (3.7%) remaining sequences did not have chromosomal location information. One-third of the *TaFBX* genomic sequences (1338) are intronless. One-quarter of the *TaFBX* (967 sequences) possesses one intron. The remaining sequences contained two or more introns, with one sequence (TraesCS5A02G409300) containing up to 21 introns.

#### 2.1.2. Genomic Distribution of TaFBX among Subgenomes, Chromosomes and Chromosomal Regions

The subgenomic distribution of the 105,200 HC genes has already been mapped onto the bread wheat pseudomolecule by the IWGSC [45] and shows that 33.60% are located on the A subgenome (35345 genes), 33.88% are on the B subgenome (35643 genes) and 32.52% on the D subgenome (34212 genes). Based on this global distribution of the wheat genes among A, B and D subgenomes, we calculated the theoretical numbers of *FBX* genes and investigated the presence of potential bias in the distribution of the 3536 *TaFBX* genes between these subgenomes. The deficit or enrichment of each subgenome was estimated (observed *TaFBX* number—theoretical *TaFBX* number). The A subgenome contained only 1031 *TaFBX* genes compared with the expected number of 1188, which represents a 4.44% deficit. The B subgenome presents a 3.39% enrichment (1318 observed vs. 1198 expected). The D subgenome appears to contain as many *TaFBX* genes as expected from the global distribution of the genes in wheat genomes (1187 observed vs. 1150 expected). This result indicates that the *TaFBX* gene distribution between A, B and D subgenomes in wheat is different from the rest of the genes (chi-squared test, *p*-value = 1.651 × 10^−4^), probably as a consequence of multiple local dynamics of the *TaFBX* genes.

**Figure 1:**
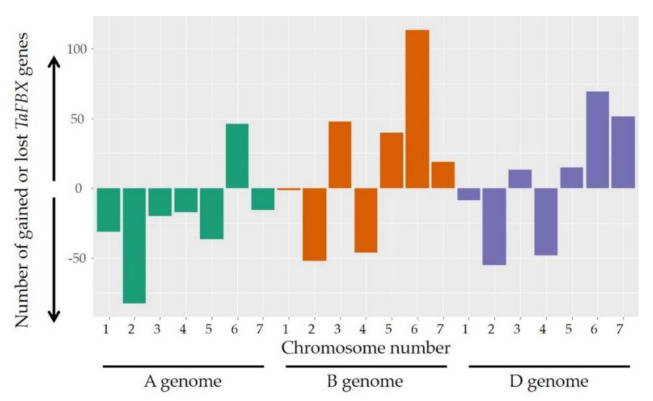
TaFBX gene excess and deficit among the 21 wheat chromosomes. The gene excess or deficit was calculated as a subtraction of the observed TaFBX number from the expected one.

We then investigated whether the distribution of the *TaFBX* genes is consistent with the global wheat gene distribution between the 21 chromosomes. The observed distribution of the 3536 anchored *TaFBX* genes on the 21 wheat chromosomes was compared with the expected one. Some chromosomes presented a deficit, while others showed a higher number of *TaFBX* genes than expected (Figure 1, Table S2). For example, while the chromosome 2A presented a deficit of 83 *TaFBX* genes, an excess of 114 genes was observed on the chromosome 6B. Regarding the D subgenome, although the total *TaFBX* gene number was found to be close to the theoretical expectations, its distribution among chromosomes is not homogeneous. For instance, the chromosome 4D presented a deficit of 48 *TaFBX* genes.

**Figure 2:**
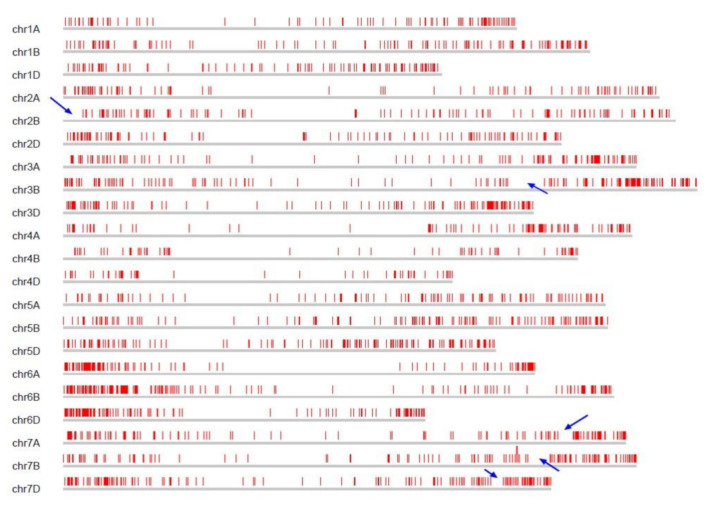
Chromosomal distribution of the *TaFBX* gene family on the bread wheat genome. Each red dash represents a *TaFBX* gene. Blue arrows highlight five chromosomal portions in the peripheral regions without any *TaFBX* genes, bordered by two regions rich in *TaFBX* genes.

To further investigate the *TaFBX* distribution on each chromosome, a scaled map was drawn using chromosomal coordinates of *TaFBX* genes (Figure 2). Their density is clearly uneven along the chromosomes. The central regions are poorly enriched in *TaFBX* genes, while the peripheral regions present a higher *TaFBX* density. Moreover, in the peripheral regions, gene clusters could be observed in some chromosomal portions, while other portions seem to be lacking in *TaFBX* genes. For example, a low *TaFBX* density is observed on the left terminal part of chromosomes 2B and 3B. Similarly, the right part of the chromosomes 7A, 7B and 7D presented a poorly *TaFBX*-containing zone, surrounded by two regions enriched in *TaFBX* genes.

**Figure 3:**
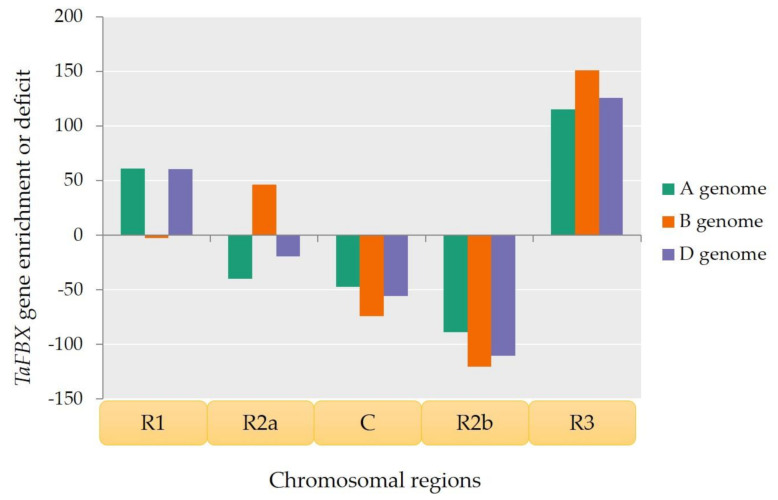
*TaFBX* gene enrichment or deficit among the five chromosomal regions of the three bread wheat subgenomes. The excess or deficit was calculated for each chromosomal region by the subtraction of the observed *TaFBX* number from the theoretical one.

To gain further insight into the unbalanced *TaFBX* gene distribution, a region-by-region analysis was performed for each chromosome, with a focus on five specific chromosomal regions originally identified by the IWGSC (2018) [45], based on their coding sequence density, transposable element content, recombination frequencies and epigenetics marks. These five regions were labeled “R1”, “R2a”, “C”, “R2b” and “R3”. A comparison of the theoretical number of *TaFBX* genes and the observed ones was performed for each of these regions for the three wheat subgenomes (Figure S1). The deficit or enrichment of each region was estimated. On the whole-genome scale, an enrichment of *TaFBX* genes appeared to be localized mainly in the R3 region, followed by the R1 region. On the contrary, the C and R2b regions showed a deficit whatever the subgenome. The results are contrasted for the R2a region; it was found enriched in *TaFBX* genes for the B subgenome but in deficit for the A and D subgenomes (Figure 3).

#### 2.1.3. Structuration of the TaFBX Genes into Subfamilies

Different subclassifications of the *FBX* family were proposed by Xu et al. (2009) (on *Arabidopsis*, rice and poplar [46]) and Jain et al. (2007) (on rice [41]) based on the large panel of supplemental domains carried by *FBX* genes. Using the same approach, a total of 17 subfamilies was established in the bread wheat *TaFBX* family, gathering gene encoding proteins with the same or similar domain organization (Table 1).

**Figure 4:**
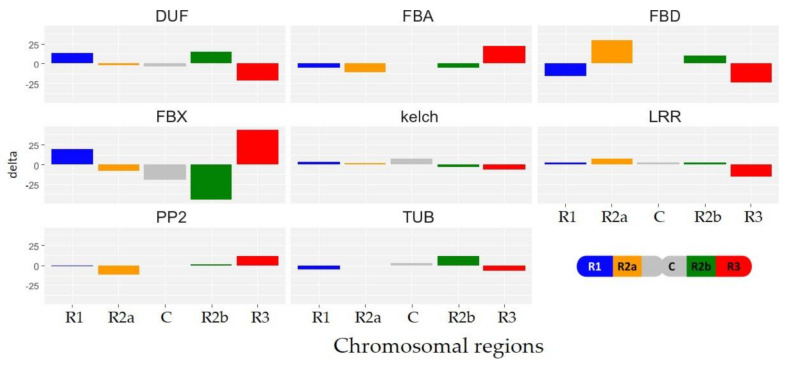
Excess and deficit of the eight major *TaFBX* gene subfamilies among the five chromosomal regions. The excess or deficit was calculated as a subtraction of the observed *TaFBX* number Figure 1. orange for R2a, grey for C, green for R2b and red for R3).

Among the five chromosomal regions, the repartition of *TaFBX* genes within the eight larger subfamilies (FBX: 2475 members, FBD: 359, FBA: 143, DUF: 234, Kelch: 203, PP2: 67, LRR: 53, and TUB: 31; regrouping 3565 *TaFBX* genes) was investigated. For each subfamily, the gene excess or deficit was evaluated in the five chromosomal regions (Figure 4 and Table S1). A selective enrichment in *TaFBX* subfamilies was observed depending on the region. The R1 region is enriched in the DUF subfamily, whereas the R2a region contains an excess of genes belonging to the FBD and LRR subfamilies. The C region presents an enrichment in the TUB and Kelch subfamilies, whereas the R2b region is enriched in the TUB, DUF and LRR subfamilies, and the R3 region in the FBX, FBA and PP2 subfamilies. Collectively, these observations suggest that *TaFBX* genes may have propagated locally, giving rise to clusters of genes belonging to the same subfamily.

#### 2.1.4. Lineage-Specific Expansion of Wheat TaFBX Genes

**Figure 5:**
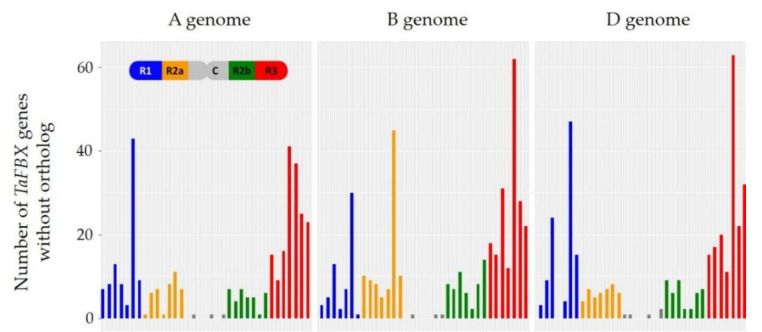
Chromosomal density of *TaFBX* genes with no orthologs identified in 18 plant species. The *TaFBX* density is presented along chromosomal regions (blue for R1, orange for R2a, grey for C, green for R2b and red for R3) for each chromosome of the A, B and D subgenomes.

Because the absolute number of *TaFBX* genes in wheat appeared significantly higher than in other plant species, we aimed at understanding the orthological relationships between wheat and other plant species. To avoid bias when searching for orthologs, we used OrthoFinder and the proteomes of 18 sequenced plant species, including monocots (as rice or *Brachypodium distachyon*), eudicots (as *Arabidopsis* or sunflower), and “basal” plants (as *Physcomitrella patens* or *Marchantia porphyria*). After filtration, 40,304 orthogroups were identified, among which 557 contained at least one wheat *TaFBX* gene (Table S3). Among the 3670 *TaFBX* genes, 2492 presented an ortholog in at least one other plant species, representing 313 orthogroups. The remaining 1178 *TaFBX* were found in 245 wheat-specific orthogroups. These *TaFBX*-with-no-ortholog genes probably constitute the hallmark of the large expansion of this family in wheat, and their chromosomal distribution is unequal (Figure 5). Whatever the wheat subgenome (A, B, or D), the distal regions R3 and R1 contained a large number of *TaFBX*-with-no-ortholog genes, whereas the C and R2b regions contained the least. The R2a region presented a low number of *TaFBX*-with-no-ortholog genes except for chromosome 6, which carried 45 genes. Considering the general *TaFBX* density across the wheat genome, the R1 and R3 regions are the strongest contributors to the set of *TaFBX*-with-no-ortholog genes. This result may suggest that novelty within this family may occur more often in the distal regions of wheat chromosomes than in their central and pericentromeric regions.

#### 2.1.5. Genomic Structuration and Duplication Fates of *TaFBX* Genes

**Figure 6:**
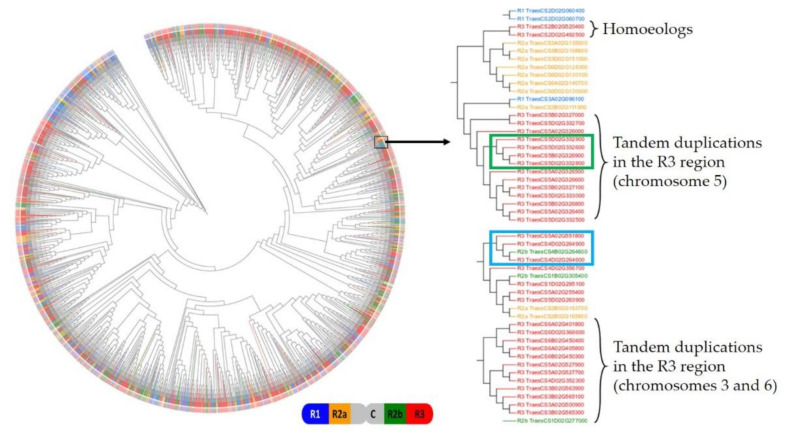
Phylogenetic tree of the 3565 TaFBX mapped on the wheat pseudomolecule. The tree was built using alignment of protein sequences. Using iTOL, each branch of this tree was annotated and colored according to the chromosomal region the gene originated from (blue for R1, orange for R2a, grey for C, green for R2b and red for R3). The branch length was not proportional to the parental degree. The right panel presents a zoom on a particular clade of the phylogenetic tree, where two specific clades are framed by a green or a blue box.

**Figure 7:**
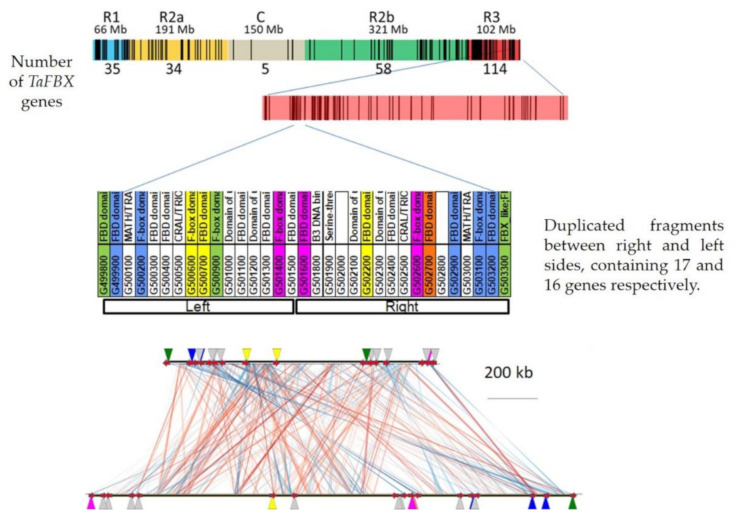
Evolutive study of a subregion of the chromosome 3B-R3 region (in red, on the top). We focused on a 3 Mb subregion, containing 33 genes including 15 *TaFBX*. The right region covers 1.09 Mb and carries 16 genes including seven *TaFBX* genes; whereas 17 genes (including eight *TaFBX*) are carried by the 1.96 Mb left region. Using the ortholog groups defined by OrthoFinder, the *TaFBX* genes belonging to the same group were colored with the same color. Their protein sequences were analyzed using Pfam and their domains were specified along with their truncated names (i.e., “G499800” means “TraesCS3B02G499800”). The red lines on the bottom part represent direct duplications and the blue ones represent reverse duplications.

To decipher the *TaFBX* gene family expansion events, we studied the relationship between *TaFBX* sequences. First, a phylogenetic tree was generated based on the alignment performed with all the mapped TaFBX proteins (3536 sequences) (Figure 6). Numerous clades are mainly composed of sequences coming from the same homoeologous group and from the same chromosomal region, as shown on the right of Figure 6. However, more complex situations were encountered. Indeed, to estimate the complex implication of different mechanisms in the local expansion of the *TaFBX* gene family, a chromosomal region rich in *TaFBX* was selected, corresponding to the R3 region of chromosome 3B that contained one of the highest excesses in *TaFBX* genes (114 *TaFBX* genes, whereas only 54 were theoretically expected). Along this region, we focused our analysis on a 3.06 Mb subregion containing 33 genes, including 15 *TaFBX*, which were found to be structured in different ortholog groups using OrthoFinder. Theoretically, in a hexaploid genome, such as in wheat, if no gene loss or gain events occur, a cardinality of 1:1:1 (one homoeolog per genome) should be observed. Using the list of homoeologous genes published by the IWGSC [45], we sought to find out whether the 33 genes anchored in this region all have a homoeolog on the A and D genomes. We found only 3 genes in homoeologous groups with a 1:1:1 cardinality, whereas 7 genes were found in groups with a cardinality of 0:1:0. Interestingly, 13 genes out of the 33 anchored in this region belonged to homoeologous groups with a cardinality of N:02:N (N = 0 or 1), which strongly suggests that there are 2 copies of each of these genes on the B genome. Collectively, it suggests that this region has undergone specific events, notably duplications, which would not have affected their ortholog counterparts on the A and D genomes. By studying the localization and orientation of the *TaFBX* genes on the pseudomolecule, this subregion was split into two parts (left and right sides, containing seven and eight *TaFBX* genes, respectively) (Figure 7). We hypothesized that they came from a local duplication event, probably, including direct and reverse duplications. To assess this hypothesis, the chromosomal sequences from both the left and the right sides were collected and compared using the YASS (blast-like) algorithm. Each fragment presenting an identity of more than 80% on at least 2 Kb was collected, totaling 386 duplicated fragments. The longest fragment reached 21.1 Kb (92% identity). Among the 386 fragments, 209 are duplicated in direct orientation and 117 in reverse orientation. Cumulatively, they represent 876 Kb of direct-duplicated fragments and 805 Kb of reverse-duplicated fragments.

To further investigations, the whole set of 33 genes (*TaFBX* and non-*TaFBX*) located on the 3 Mb subregion were also studied. As expected, 14 genes were found to contain the F-box domain (IPR001810) in the N-terminal and the FBD domain (IPR006566) in the C-terminal of their protein sequences. Six other proteins contained only the FBD domain, and one protein contained only the F-box domain. The 12 remaining proteins contained various domains, like MATH/TRAF domain (IPR002083) for two of them or Domain of Unknown Function DUF1618 (IPR011676) for four of them.

**Figure 8:**
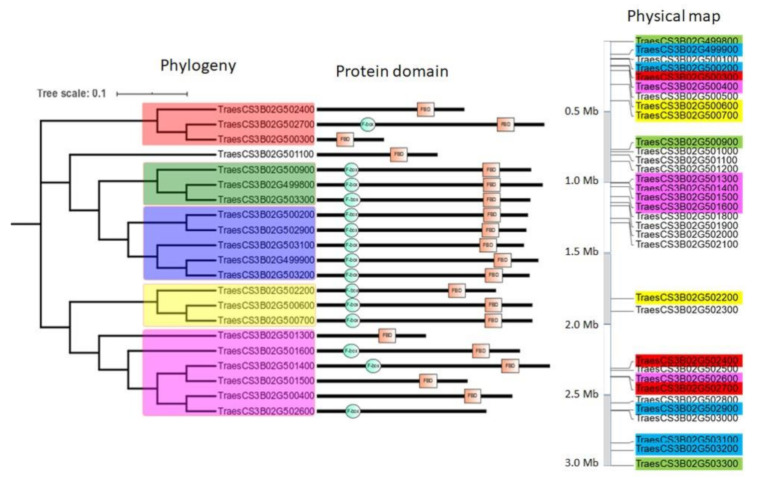
Phylogenetic tree of the 21 genes containing a FBD domain (red box) and/or a F-box domain (green circle), and their physical distribution on a 3 Mb part of the chromosome 3B-R3 region. The different clades are delimited by various colors, reported onto the physical map to identify their spatial organization.

Because the FBD domain is often associated with the F-box domain in proteins, we focused on the 21 proteins containing either the FBD domain alone, the F-box domain alone, or both (Figure 8). Among the five clades, we identified three clades (blue, green and yellow) composed of genes coding for F-box and FBD domain-containing proteins. The two last clades are a patchwork between genes coding for proteins containing one or both of the F-box and the FBD domains. Collectively, these findings indicate that this region may have experienced multiple loss or gain of domains, probably through unequal crossing-over and local duplications. However, whether these truncated proteins are still capable of interacting with other proteins (in particular, the SKP1 subunit of the target protein) must be experimentally verified.

### 2.2. TaFBX Family Expression Pattern

*FBX* genes are known to be involved in various biological processes in plants, such as growth and stress response. We investigated the expression pattern of this large family in bread wheat, during embryogenesis/grain development and in response to heat and drought stress. For this purpose, we collected transcriptomic data from public repositories and extracted the ones corresponding to the *TaFBX* genes for specific analysis.

#### 2.2.1. *TaFBX* Expression during Wheat Embryogenesis and Grain Development

An exhaustive analysis of the transcriptome dynamic during embryogenesis and endosperm development was recently published [47]. In this study, the authors identified 39,173 DEGs at different developmental stages. Among those DEGs, we retrieved 1206 *TaFBX* genes and collected their chromosomal coordinates in order to classify them according to their chromosomal region (Table S4). The observed *TaFBX* number of each of these regions was compared with the theoretical ones (Table 2). Clearly, the *TaFBX* distribution is not equal between observed and theoretical estimations across the whole of the *TaFBX* family. Indeed, the interstitial regions are overrepresented (particularly the R2b region), whereas the R1 and R3 distal regions are underrepresented. These results emphasize the fact that, although the distal regions contained an absolute high number of *TaFBX* DEGs during wheat grain development, their relative contribution is less important than the interstitial regions (R2a, C and R2b).

#### 2.2.2. *TaFBX* Gene Expression in Response to Heat and Drought Stress

To study the involvement of *TaFBX* genes in the wheat response to heat and drought stress, RNAseq data generated by Liu et al. (2015) [48] were used (accession number SRP045409). In their experiment, 7-day wheat seedlings were submitted to thermal stress, drought or a combination of both for 1 h or 6 h for each treatment. As done previously, only the HC genes were retrieved, and the counts of all of the isoforms were cumulated for each gene, leading to a 107,891 gene set. After filtration, 55,801 genes were found expressed, containing 1298 *TaFBX* sequences. By comparing stressed and control samples, a total of 28,989 DEGs were identified, among which 525 were *TaFBX* coding sequences (Table S5).

Observed and theoretical distribution of *TaFBX* genes responding to abiotic stress were analyzed by considering their location in the chromosomal regions (Table 3). Similar to the results observed during wheat embryogenesis, the chromosomal regions did not contribute equally to the set of differentially expressed *TaFBX.* A higher number of *TaFBX* DEGs were noted in the R2b, R2a and C interstitial regions compared with the R1 and R3 distal regions. The R2b region is the largest contributor of *TaFBX* DEGs, with 160 observed *TaFBX* genes compared with 95 theoretically expected (representing an excess of 65 genes).

The distribution of *TaFBX* DEGs, both during embryogenesis/grain development and in response to abiotic stress, indicated that, while the distal R3 region displayed an absolute high number of DEGs, its relative contribution is modest considering its total *TaFBX* content.

## 3. Discussion

### 3.1. FBX Family Size Is Larger in Wheat Than in Other Known Plant Species

The FBX family is one of the largest gene families in plants, but only a few members have been fully characterized. A wide and complete identification of the *FBX* gene family is a mandatory step before the characterization of their biological functions. Hence, far, several genomes were explored to identify the *FBX* gene family. Among eukaryotes, this family is usually composed of less than 100 members (human, mouse, *Saccharomyces* or *Drosophila* [28,29]) with a maximal number of 326 FBX in *C. elegans* [24]. In the plant kingdom, however, an FBX family with an order of magnitude from a hundred to a thousand gene members is often observed (rice, *Arabidopsis*, maize, moss, or poplar [25,30]). Through their essential role in targeting specific proteins, they are involved in a wide range of physiological processes, including developmental and adaptive ones, in response to environmental stress [14,41,49,50,51,52,53,54,55,56,57,58,59,60,61,62,63,64,65,66,67].

While this manuscript was in its final stage, Hong et al. (2020) reported that the bread wheat genome contains 1796 *TaFBX* genes [43]. Using the same RefSeq v1.1 version of the annotated wheat genome released by the IWGSC [45], we identified 3670 *TaFBX* genes, which are twice larger. This discrepancy between the two results could be explained by the validation and manual curation process of the putative sequences identified. Indeed, while both Hong et al. (2020) and we identified roughly the same initial number of *TaFBX* putative genes using HMMR programs (around 4000 unique genes), they may have used more restrictive filters and only the Pfam database to identify their final set of *TaFBX* genes. In addition, those authors indicated that they removed identical sequences. In our paper, we used InterProScan and interrogated the InterPro database that integrates signatures from 13 different databases [68]; this may have led to retrieving more sequences. Furthermore, the annotation of the whole proteome of wheat by the IWGSC identified 3749 unique proteins containing the F-box domain (IPR001810). Finally, in the set identified in our paper, we retrieved sequences coding for essentials proteins, such as putative receptors for hormones that were not retrieved by Hong et al. (2020) [43], for instance Auxin-signaling (8 TaFBX, TraesCS7D02G177500, TraesCS7B02G081200, TraesCS1D02G099900,TraesCS1A02G091300,TraesCS1B02G119100, TraesCS5B02G280100, TraesCS5A02G281100, TraesCS5D02G288000), jasmonate perception and signaling (TraesCS3D02G360400, TraesCS3B02G399200, TraesCS1A02G279100, TraesCS1B02G288100, TraesCS1D02G278400, TraesCS3A02G367500) or abscisic acid signaling (TraesCS3A02G331200,TraesCS3B02G361400,TraesCS1B02G244300,TraesCS1A02G229800,TraesCS1D02G232200). Collectively, these arguments suggest that the number of *TaFBX* genes (i.e., 1796) reported by Hong et al. (2020) [43] is likely an underestimation of the actual number of *TaFBX* in the wheat genome and, while the number of 3670 genes reported here could vary somewhat, it is likely more representative of the actual size of this family.

### 3.2. FBX Gene Family Has Experienced Local Expansion through Retrotransposition, Tandem and Interchromosomal Duplications

Various mechanisms have been proposed to explain the expansion of gene families in plants. They included, for instance, retrotransposition, unequal crossing-over, duplicated DNA transposition, and polyploidization [69,70,71,72]. Once duplicated, the gene could retain its original function, acquire a new function (neofunctionalization) or even lose any identifiable function (pseudogenization) [73].

Among the *TaFBX* family identified here, 1338 (36.5%) are intronless and probably correspond to processed genes that have been duplicated through the retrotransposition phenomenon [74]. This percentage is close to those observed for FBX in other species (chickpea, 34% [31] and rice 41% [41]) and could partially explain the global family expansion observed in wheat in comparison with other species.

Moreover, the phylogenetic tree (Figure 6) suggests that other mechanisms would be responsible for the TaFBX family expansion. First, numerous clades are mainly composed of genes physically close to each other on the pseudomolecule. This result supports the hypothesis of tandem duplications as one of the main mechanisms of the *TaFBX* family expansion. Some of these duplications include three members from one homoeologous group, which supports the hypothesis of an ancestral duplication acquired by the common ancestor of the three subgenomes. In some other cases, only one homoeolog appears to have been duplicated once or more, leading to the generation of extra copies of this gene. One example is given in the green box of Figure 6, showing one *TaFBX* gene (TraesCS5D02G332800) that may have been duplicated to give two inparalogs (TraesCS5D02G332900 and TraesCS5D02G332600). More complex situations can be encountered (blue box of Figure 6). In this case, if the percentage of sequence identity is the criterion to identify homoeologous genes, TraesCS4B02G264600 would be the homoeolog of TraesCS4D02G264600. Alternatively, the B copy and the two D copies could come from duplication between non-homoeologous loci after a double-strand break, as has been observed for other genes [75]. This hypothesis could be supported by the presence of another copy in the same clade, carried by chromosome 5 of the A subgenome (Tres5A02G551800). Glover et al. (2015) [75] suggested that interchromosomal duplications do happen in the wheat genome after double-stranded DNA break repairs and contribute significantly to the duplication of genes in non-syntenic regions (anchored on different chromosomes). It also has been suggested that these small-scale duplications may explain the expansion of the NAC transcription factors family in wheat [38,76].

To go deeper into the understanding of the duplication pattern that could have occurred during the *TaFBX* gene evolution and expansion, we studied a particular subregion of the R3 region of the chromosome 3B. Indeed, a phylogenetic tree regrouping *TaFBX* anchored in this subregion was constructed (Figure 8). Two clades were found to contain either FBD domain, F-box domain or both. This could be explained by sequence shuffling that occurred after nucleotidyl insertions/deletions phenomena. These events generated proteins of various lengths and, in some cases, without the F-box motif (i.e., TraesCS3B02G502400) or without the FBD motif (i.e., TraesCS3B02G502600). On chromosome 3B, two *TaFBX* genes, localized side by side and within the same clade (TraesCS3B02G500600 and TraesCS3B02G500700), present almost 100% identity in their protein sequences (except for 14 amino acids in their C-terminal regions), suggesting duplication of one copy, followed by limited sequence variation. A third gene (TraesCS3B02G502200), physically distant from the two others, is present in the same clade. The extra-copy probably originated from a duplication of one of the other genes, followed by insertion of four nucleotides and deletion of 276 nucleotides in its C-terminal region. As a result, the three copies share the F-box domain, which is necessary for interaction with the SKP1 sub-unit; however, they diverged in the C-terminal region, which is involved in the interaction with putative substrates. In a general manner, the whole subregion seems to be organized as a mirror image, with the alignment of its left reflecting that of its right, highlighting evolutive resemblances. Interestingly, when considering the alignment of the left side with the right one, we noted that the direct and reverse duplications do not occur in a single contiguous block but with a “direct-reverse” alternation pattern, suggesting multiple and complex small-scale duplication events (Figure S3).

Collectively, these observations indicate that multiple rounds of duplications through retrotransposition, tandem and small-scale duplications, combined to sequence diversification through insertions and deletions, are responsible for the large and diversified repertoire of *TaFBX* genes in wheat.

### 3.3. The TaFBX Family Structured into Subfamilies Is Unequally Expanded in Wheat

It is known that the *FBX* characterized genes do carry other domains than the F-box domain that are localized in their C-terminal regions [41]. However, here, we found that 67% of the *TaFBX* genes do not have any additional known domains. This result is in accordance with the protein structure that has been observed in cotton and in wheat with, respectively, 54% and 76% of the FBX containing only the F-box domain [32,43]. A similar percentage was observed by Jain et al. (2007) [41] in the rice *FBX* family. In contrast, only 14% and 30% of the *FBX* genes were identified without other known domains in *Arabidopsis* and chickpea, respectively [31,77]. The second most abundant subfamily was found to be the FBD subfamily in our study, such as in Hong et al. (2020) [43], while this is the Kelch domain in cotton [32].

Analysis of the remaining *TaFBX* genes showed the presence of supplemental domains, such as FBD, DUF, Kelch, FBA, etc., localized in the C-terminal region of their sequence and used for their subclassification into 17 subfamilies. Among those wheat *TaFBX* subfamilies, we identified 67 genes carrying the phloem protein 2-like domain (or PP2, IPR025886). The latest examination of the *Arabidopsis*, poplar and rice genomes [41,46], as well as other plant species [30], did not reveal any FBX protein with this domain. More recently, eight FBX genes carrying the PP2 domain have been identified in chickpea [31]. It has been suggested that, through evolution, some PP2 proteins (typically associated with phloem functions) have acquired the FBX domain and gained the capability to interact with glycoproteins, playing a role in protein degradation [31,78].

### 3.4. The FBX Gene Distribution Is Not Homogeneous in the Wheat Genome

Globally, the *TaFBX* gene distribution was revealed at different genomic scales (subgenomic, chromosomal and chromosomal region scales). The potential deficit/enrichment was estimated by calculating the theoretical (expected) number of *TaFBX* genes, considering the global gene distribution at each studied scale. A few members of the *TaFBX* family (3.7%) are unanchored on the bread wheat chromosomes. This finding is in agreement with the results observed for other gene families in wheat, such as the *TaNAC* family, in which 2% of the genes have been found to have no chromosomal location [38]. What is more, the anchored *TaFBX* genes are not regularly dispatched among the three subgenomes, on the seven chromosomes of each subgenome, nor in the five chromosomal regions of each chromosome. By comparing the observed subgenomic distribution to the expected one, we showed that the A subgenome displays a deficit in *TaFBX*; the B presents an enrichment, whereas the D is rather balanced. However, this balanced distribution is not homogenous among the seven chromosomes. For example, the chromosome 4D contains fewer genes than expected.

### 3.5. FBX Gene Distribution Is Not Homogeneous among the Five Chromosomal Regions of the Bread Wheat Genome

The wheat genome seems to present a preferential anchorage of the *TaFBX* genes in certain regions of the chromosomes. The resulting unequal distribution suggests a key role of proximal and tandem duplications in the local expansion of the *TaFBX* subfamilies. This hypothesis is supported by the observation that the R1 and R3 regions are enriched in *TaFBX*-with-no-ortholog genes in comparison with the R2a, R2b and C regions. These genes can be named “lineage-specific”. Considering the lack of orthology as a reflection of divergence in terms of coding sequences between plant species, the R1 and R3 regions would be the preferential regions for innovation in their coding sequences, with a strong and active divergence. The large accumulation of *TaFBX*-with-no-ortholog genes in the R1 and R3 regions is consistent with the fact that these regions contain the highest number of repeated sequences and recombination frequency compared with the other chromosomal regions [45]. Furthermore, the five regions of each of the seven chromosomes do not present the same *TaFBX* distribution. For example, chromosome 1 is particularly enriched in *TaFBX* genes in the R3 region, whereas chromosome 6 presents a *TaFBX* enrichment in its R1 region. This result suggests that different events (such as duplication, insertion, deletion, mutation, etc.) must have affected the seven chromosomes independently.

Moreover, a similar profile can be shared between two or three homoeologous chromosomes, suggesting that this profile can be considered as the (likely) ancestral profile present before the wheat hexaploidization. For example, the same *TaFBX* excess/deficit profile can be observed in the C, R2b and R3 regions of the chromosomes 1A, 1B and 1D, but the profile differs in the R1 and R2a regions of the chromosome 1A versus chromosomes 1B and 1D. This observation suggests that the ancestral state was conserved in the chromosomes 1B and 1D, but the chromosome 1A must have experienced a different fate leading to a *TaFBX* gain in its R1 region and *TaFBX* loss in its R2a region (Figure S2).

### 3.6. Differentially Expressed TaFBX Are Concentrated in the R2b Region

Classically, transcriptome analyses are useful resources for global gene expression analysis. This kind of upstream work is often used to predict putative functions and then identify key genes in plant physiology. Here, we wanted to find out whether each chromosomal region contributed equally in terms of gene expression. For this purpose, we identified and localized *TaFBX* DEGs in the chromosomal regions. We chose two main physiological processes as examples: embryogenesis and response to heat and drought stress, based on already published RNAseq data.

First, using the transcriptome analysis realized during seven stages of embryogenesis and endosperm development [47], we identified 1206 *TaFBX* genes with a differential expression in the embryo, endosperm, and/or pericarp (Table S4). Secondly, in response to abiotic stress, 525 *TaFBX* DEGs were identified from the data of Liu et al. (2015) [48] (Table S5). A focus on their chromosomal anchorage revealed that their distribution pattern along the wheat genome did not match with the expected one from the whole *TaFBX* gene distribution. For both datasets, we found that, although the distal chromosomal regions (R1 and R3) were richer in genes than the central (C) and interstitial regions (R2a and R2b), the latter being richer in *TaFBX* DEGs than expected during embryogenesis and in response to abiotic stress. Cumulatively, the R2a, R2b and C regions contributed 16.3% more *TaFBX* DEGs than expected during embryogenesis and 50.9% more in response to abiotic stress. The most significant gap between theoretical and observed *TaFBX* gene differential expression was noticed in the R2b region in response to abiotic stress, with 68% more DEGs than expected. To explain these observations, one hypothesis puts forward that the interstitial regions (R2a, C and R2b), which contain more conserved genes (IWGSC, 2018), may contribute significantly to basic and essential biological processes, such as embryogenesis and in response to environmental stress, two processes, which require stability during evolution. In contrast, the distal chromosomal regions (R1 and R3), which are more prone to duplication and recombination events [79], may contribute by providing innovative functions to adapt to particular conditions. This hypothesis was already suggested by the members of the IWGSC (2018) [45], Ramírez-González et al. (2018) [80] and Schilling et al. (2020) [37]. They observed that the MADS-box MIKC-type transcription factors, which are involved in highly conserved developmental functions, are preferentially located in the central chromosomal segments and that they belong to smaller subclades. In contrast, larger subclades contain genes mostly located in the distal regions. Those genes are mostly involved in the adaptation to environmental cues, such as the *FLC-like* gene, which is involved in flowering time [37]. Thus, we hypothesize that the set of *TaFBX* originating from the central regions (R2a, C and R2b), and differentially expressed, constitute the core of the conserved and general wheat FBX genes involved in embryogenesis and in response to abiotic stress, while those originating from the R1 and R3 regions may provide a subtle, but nevertheless essential, contribution to the wheat’s ability to adapt to its environment.

## 4. Materials and Methods

### 4.1. Survey of TaFBX in the Wheat Sequence

The FBX proteins were retrieved from the protein database of the IWGSC (IWGS RefSeq v1.1, cv *Chinese Spring*) [45] by using the HMMER2 program implemented in Unipro UGENE 1.25 [81]. The FBX and FBX-like motifs (PF00646, PF12937, PF13013, and PF15966), downloaded from Pfam, were used as queries. The initial set of genes identified by HMMER2 were subjected to InterProScan analysis and manual curation.

### 4.2. Phylogenetic Analysis of the FBX Family

As our aim was to analyze the relationships between sequence relatedness and the chromosomal localization of the *TaFBX* genes, we only retained the *TaFBX* sequences that were mapped onto the wheat genome (i.e., 3536 sequences). The complete amino acid sequences were aligned using MAFFT algorithms (https://mafft.cbrc.jp/alignment/server/ date of access: 6 May 2020 [82]). The alignment was then used without further manual correction to construct an approximately maximum-likelihood phylogenetic using FastTree algorithms (FastTree 2.1.11 [83]) with default parameters. The tree obtained was then rendered using iTOL (https://itol.embl.de/, date of access: 6 May 2020 [84]).

### 4.3. Chromosomal Distribution of TaFBX Coding Genes

The genomic coordinates of each *TaFBX* gene (start and end positions) were extracted from the GFF file provided by the IWGSC [45]. These coordinates were then used to draw the chromosomal distribution of the *TaFBX* genes among the 21 wheat chromosomes using KaryoploteR [85]. These same coordinates were also used to distribute the genes between the R1, R2a, C, R2b and R3 regions as defined by the IWGSC [45]. Finally, since each of these regions contains a known total number of genes, we used this information to estimate the expected number of *TaFBX* genes per region, assuming that the chromosomal distribution of *TaFBX* followed the same distribution as the rest of the wheat genes [45]. The goodness of fit of the observed number of *TaFBX* per region to that expected was tested using the chi-squared test.

### 4.4. Evolutionary Study of the R3 Region of the Chromosome 3B

To gain insight into the mechanisms leading to the local expansion of *TaFBX* genes, the R3 region of chromosome 3B was selected for its high-density in *TaFBX* genes. A focus was made more specifically on the subregion spanning from 759562499 to 762622854 (i.e., about 3.06 Mb). The genomic sequence of this region was downloaded and analyzed using YASS, a blast-like algorithm [86]. Any two non-overlapping sequences, presenting a minimum of 80% identity covering a minimum of 2 Kb, were considered duplicated. Overall, this approach identified two duplicated blocks whose coordinates were from 759562499 to 760651332 for the first one (approximately 1.09 Mb) and from 760655083 to 762622854 for the second one (approximately 1.96 Mb).

### 4.5. Expression of TaFBX Genes

#### 4.5.1. *TaFBX* Gene Expression during Embryogenesis and Grain Development

To identify the *TaFBX* whose expression varied during development, we extracted the list of differentially expressed F-box coding genes from Xiang et al. (2019) [47]. In this study, the authors used seven stages of embryogenesis (from the two-cell stage to the mature stage), two endosperm stages, and the pericarp, comparing their transcriptome programming using RNAseq. In total, they identified 39,173 DEGs.

#### 4.5.2. *TaFBX* Gene Expression in Response to Heat and Drought Stress

Raw data corresponding to the experiments described in Liu et al. (2015) [48] was downloaded from the www.wheat-expression.com (date of access: 6 May 2020) platform and analyzed using the LIMMA package [87]. Weakly expressed genes were first removed by filtration of raw expression data corresponding to the entire wheat genome (genes were retained if they were expressed at a counts-per-million > 0.5 in at least two samples). TMM and Voom methods were then used to normalize the expression of retained genes. Their expressions were compared between samples, and genes that displayed an absolute log fold-change >1 along with an adjusted *p*-value < 0.05 were deemed DEGs. A total of 28,989 genes were identified as DEGs, among which 525 corresponded to F-Box coding genes.

Finally, we focused on detecting any potential bias in the chromosomal distribution of the *TaFBX* differentially expressed either during embryogenesis or in response to heat and drought stress; the number of *TaFBX* DEGs observed per region was compared with that expected using a chi-squared test (see above, chromosomal distribution section).

## 5. Conclusions

Using the latest assembly of the wheat genome, we retrieved 3670 *TaFBX* genes. The *FBX* in wheat appears to be a particularly large multigenic family in comparison with most plant species, suggesting an expansion through retrotransposition (processed genes), tandem and small-scale duplications. We showed that their distribution is not homogeneous between A, B and D wheat subgenomes, nor between the chromosomes and the five different chromosomal regions of each chromosome (R1, R2a, R2b, C and R3). Moreover, in a chromosome particularly enriched in *TaFBX*, this enrichment is concentrated in its distal regions. However, despite being the richest regions in *TaFBX* genes, the distal regions are not the most significant reservoir of *TaFBX* DEGs during wheat embryogenesis (1206 *TaFBX* genes) or in response to heat and drought stress (525) compared with the expected numbers that could have been obtained when considering the gene distribution in the whole-genome. This observation may suggest that the central regions of wheat chromosomes contain fewer genes but that they may contribute more significantly to essential biological processes. On the other hand, the distal regions contain more genes but seem to contribute less than expected when considering their gene densities alone.

## Figures and Tables

**Table 1:** Distribution of the *TaFBX* genes among 17 subfamilies, in view of the presence of the F-box domain and other FBX-specific domains. The FBX-only subfamily carries only the F-box domain. The “Other” subfamily contains *TaFBX* genes with rarely observed domains.

Subfamily (Subdomain Contained by their TaFBX Members)	Number of TaFBX Genes
ACTIN (Plant actin-related protein 8, IPR030071)	3
ARM (Armadillo, IPR000225)	10
DUF (Domain unknown function DUF295)	234
FBA (F-box associated domain, IPR007397)	143
FBD (FBD domain, IPR006566)	359
FBX-only (F-box domain, IPR001810)	2475
Jmjc (Jmjc domain, IPR003347)	9
Kelch (Galactose oxidase/kelch, beta-propeller, IPR011043 or Kelch-type beta propeller, IPR015915)	203
LRR (Leucine-rich repeat, IPR001611)	53
LysM (LysM domain, IPR018392)	3
Other (various domains described in Table S1)	41
PP2 (Phloem protein 2-like, IPR025886)	67
QAD (Quinoprotein amine dehydrogenase, beta chain-like, IPR011044)	17
SEL1 (Sell-like repeat, IPR006597)	3
TUB (Tubby, C-terminal, IPR000007)	31
WD40 (WD40 repeat, IPR001680)	14
zf_MYND (Zinc finger, MYND-type, IPR002893)	5
Total	3670

**Table 2:** Distribution of TaFBX DEGs during embryogenesis and endosperm development, according to their anchorage on chromosomal regions (X-squared test = 20.938, df = 5, *p*-value = 0.0008322).

Chromosomal Region	Number of Genes in the Whole TaFBX Family	Theroretical *TaFBX* DEGs		Observed *TaFBX* DEGs	
		Number	%	Number	%
R1	643	211.30	17.5	176	14.6
R2a	637	209.32	17.4	236	19.6
C	76	24.97	2.1	34	2.8
R2b	662	217.54	18	255	21.2
R3	1518	498.83	41.4	486	40.4
Unanchored	134	44.03	3.7	17	1.4
Total	3670	1206	100%	1206	100%

**Table 3:** Comparison of the observed and theoretical distributions of the *TaFBX* genes responding to abiotic stress according to their anchorage in the chromosomal regions (X-squared test = 46.212, df = 5, *p*-value = 8.222 × 10^−9^).

Chromosomal Region	Theroretical *TaFBX* DEGs		Observed *TaFBX* DEGs	
	Number	%	Number	%
R1	92	17.5	45	8.6
R2a	91.1	17.4	115	21.9
C	10.9	2.1	22	4.2
R2b	94.7	18	160	30.5
R3	217.2	41.4	169	32.2
U	19.2	3.7	14	2.7
Total	525	100%	525	100%

## Data Availability

The data that support the findings of this study are available from the corresponding author upon reasonable request.

## Supplementary Materials

Supplementary Materials can be found at https://www.mdpi.com/1422-0067/22/6/3111/s1.

## Abbreviations

DEG	Differentially Expressed Gene
FBX	F-box proteins
Ta	*Triticum aestivum*, bread wheat
HC	High Confidence
