# Supplementary figures and images for: The Landscape of the Genomic Distribution and the Expression of the F-Box Genes Unveil Genome Plasticity in Hexaploid Wheat during Grain Development and in Response to Heat and Drought Stress
Source: Int J Mol Sci. 2021 Mar 18;22(6):3111. doi: 10.3390/ijms22063111 (PMC8002965; doi:10.3390/ijms22063111)

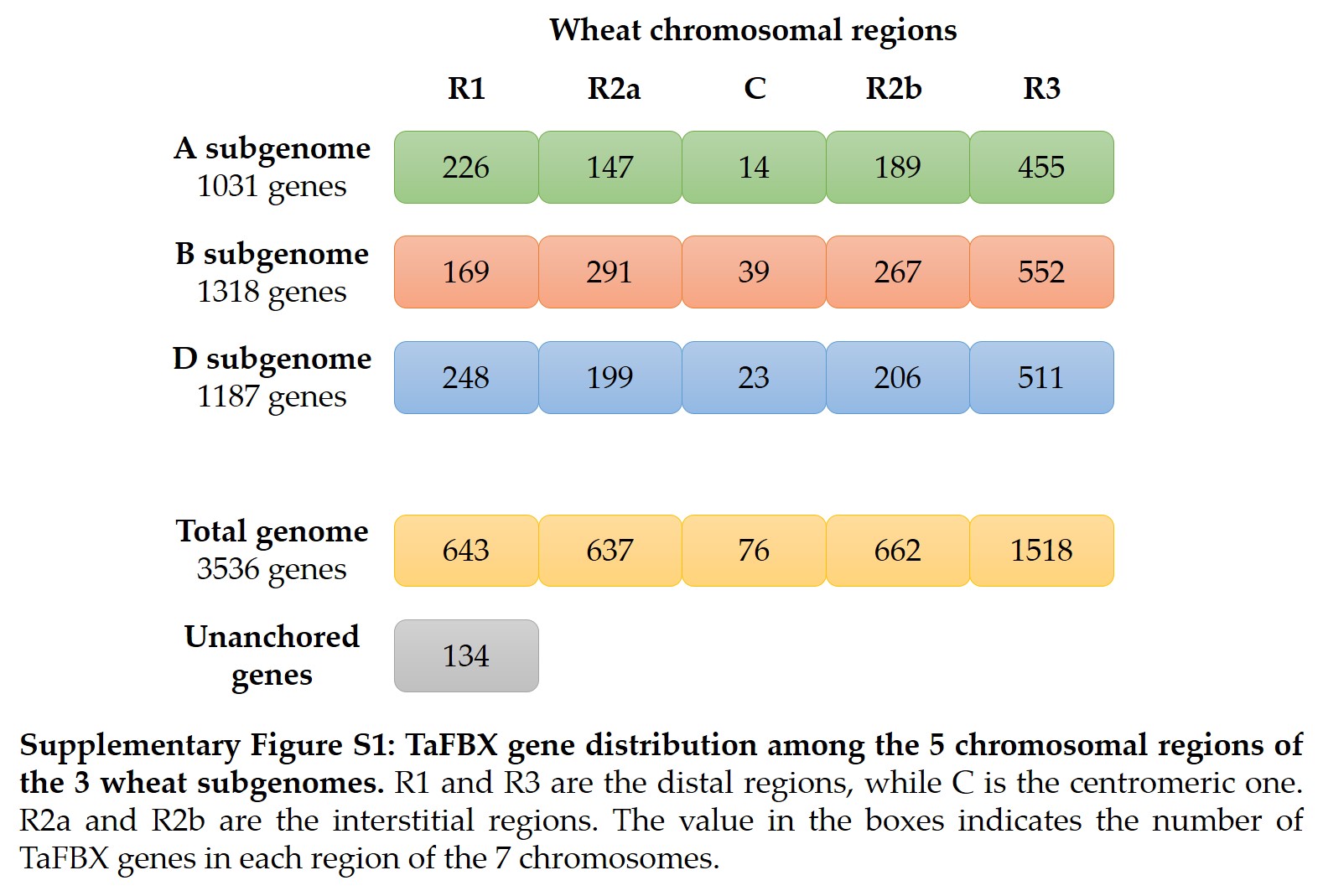

Supplement: Supplementary file 1 [file ijms-22-03111-s001.zip › Supplemental Data/S1_Figure.jpg]

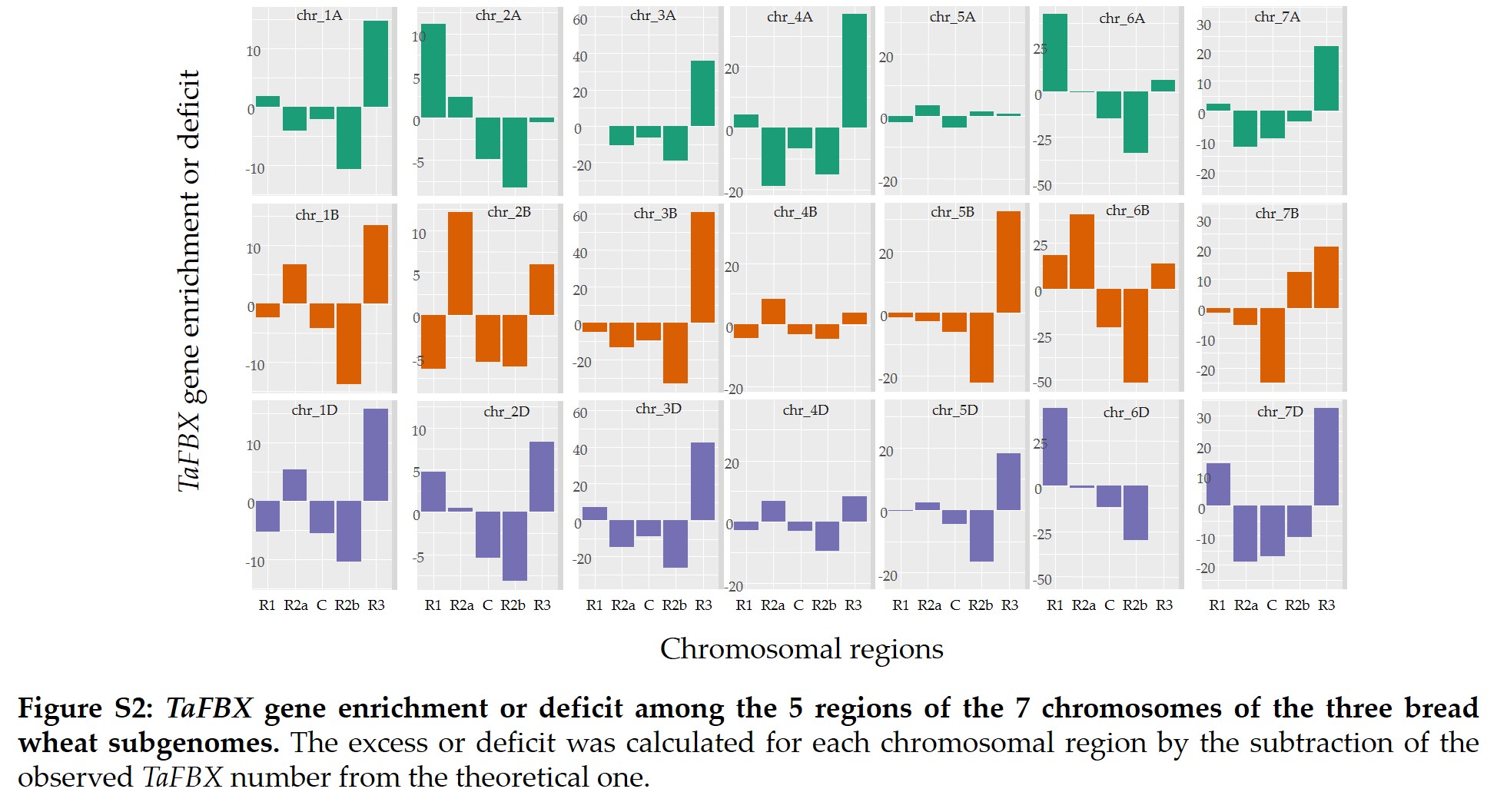

Supplement: Supplementary file 1 [file ijms-22-03111-s001.zip › Supplemental Data/S2_Figure.jpg]

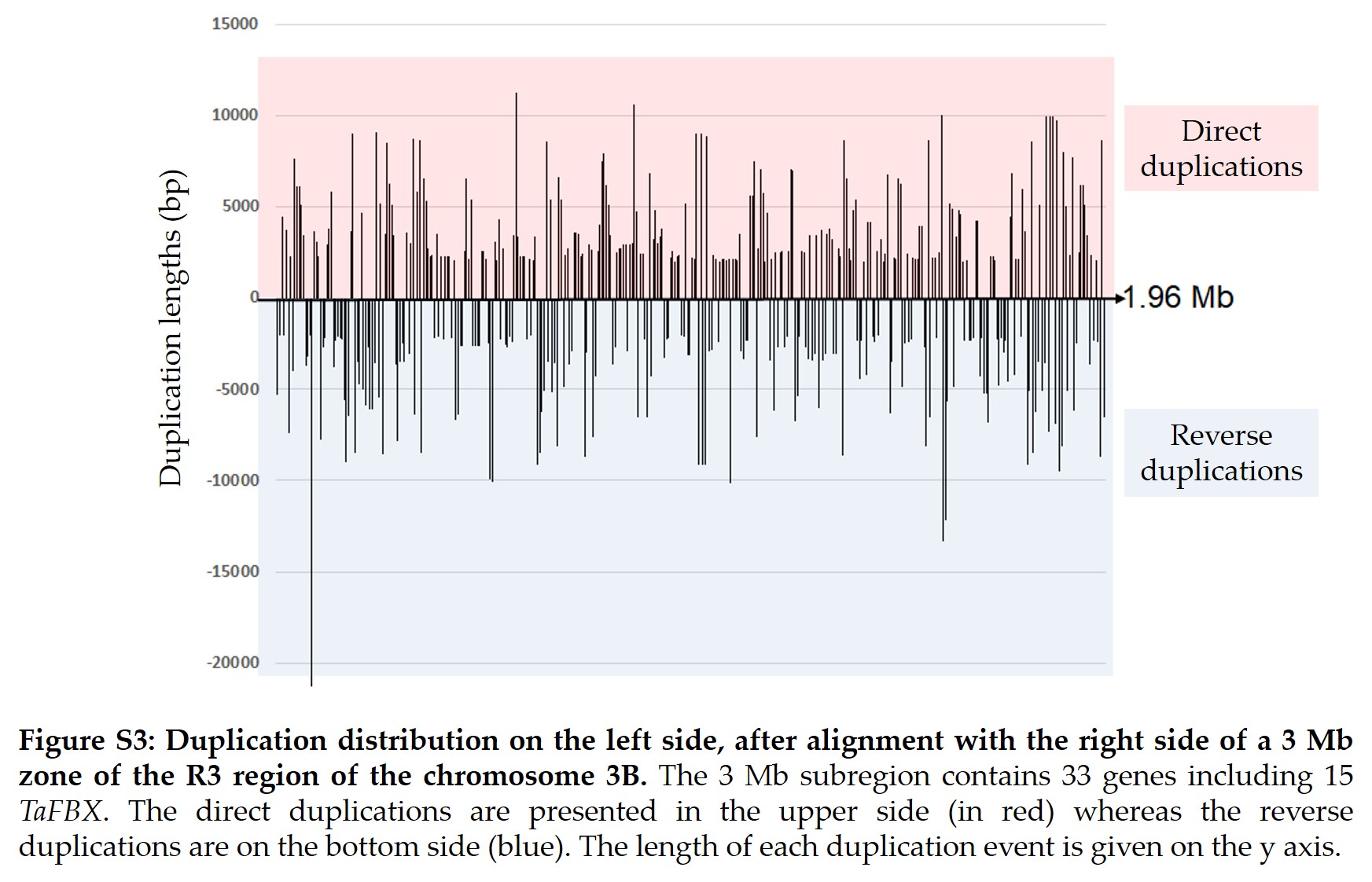

Supplement: Supplementary file 1 [file ijms-22-03111-s001.zip › Supplemental Data/S3_Figure.jpg]
